# Sensitivity of the Breastfeeding Motivational Measurement Scale: A Known Group Analysis of First Time Mothers

**DOI:** 10.1371/journal.pone.0082976

**Published:** 2013-12-31

**Authors:** Janine Stockdale, Marlene Sinclair, George Kernohan, Evie McCrum-Gardner, John Keller

**Affiliations:** 1 School of Nursing and Midwifery, University of Ulster, Belfast, Northern Ireland; 2 School of Nursing, University of Ulster, Belfast, Northern Ireland; 3 Clinical Research Support Centre, Belfast, Northern Ireland; 4 Instructional Systems Faculty, Florida State University, Tallahassee, Florida, United States of America; London School of Economics, United Kingdom

## Abstract

Breastfeeding has immense public health value for mothers, babies, and society. But there is an undesirably large gap between the number of new mothers who undertake and persist in breastfeeding compared to what would be a preferred level of accomplishment. This gap is a reflection of the many obstacles, both physical and psychological, that confront new mothers. Previous research has illuminated many of these concerns, but research on this problem is limited in part by the unavailability of a research instrument that can measure the key differences between first-time mothers and experienced mothers, with regard to the challenges they face when breastfeeding and the instructional advice they require. An instrument was designed to measure motivational complexity associated with sustained breast feeding behaviour; the *Breastfeeding Motivational Measurement Scale*. It contains 51 self-report items (7 point Likert scale) that cluster into four categories related to perceived value of breast-feeding, confidence to succeed, factors that influence success or failure, and strength of intentions, or goal. However, this scale has not been validated in terms of its sensitivity to profile the motivation of new mothers and experienced mothers. This issue was investigated by having 202 breastfeeding mothers (100 first time mothers) fill out the scale. The analysis reported in this paper is a three factor solution consisting of value, midwife support, and expectancies for success that explained the characteristics of first time mothers as a known group. These results support the validity of the BMM scale as a diagnostic tool for research on first time mothers who are learning to breastfeed. Further research studies are required to further test the validity of the scale in additional subgroups.

## Introduction

Breastfeeding has immense public health value for mothers, babies and society. However, national data demonstrates that many women, particularly first-time mothers, within the UK [1], Ireland [2] and USA [3] will stop breastfeeding within the first few weeks. Understanding why first time mothers are more likely than experienced mothers to stop breastfeeding is essential to the development and design of effective breastfeeding promotion and support programmes. First-time mothers who stop breastfeeding tend to be younger, less educated and single [1]–[2]. In addition, they are more likely than experienced mothers, to be exposed to breastfeeding promotional activity, which although is designed with women's best interests in mind, has been accused of being over enthusiastic in its attempt to counteract the influences of a bottle feeding culture [4].

However, to say that breastfeeding promotion and support has been over enthusiastic is too general and imprecise. More specifically, while there has been an increase in the use of applied psychological theory to explain breastfeeding behaviour, for example self-efficacy [5], intrinsic and extrinsic motivation [6], attitudes, social norms and perceived control [7]–[9], self-identity [10] and goal setting [11], limited information is known about how these key motivational factors influence the experience of first time mothers and their support requirements. That is, if we do not know how to measure the key motivational differences of first time mothers, we cannot design the learning environment in order to meet the psychological learning needs of this group of women.

### Understanding human motivation

A major factor underpinning human behaviour is understanding what motivates an individual to change their behaviour in a positive direction. A key theorist, who has been working in this area for over 40 years, proposes that motivation to engage in a behaviour *“assumes people are motivated to engage if the activity is perceived to be linked to satisfaction of a personal need (value aspect) and if there is a positive expectancy for success (expectancy/learning aspect* [12] (p 3). Therefore, when a motivational imbalance exists and a woman lacks value for breastfeeding, and/or doesn't believe she can succeed, she is unlikely to persist with learning *how* to breastfeed. Evidence to support this hypothesis can be found in research studies that have used ‘expectancy-value theories’ such as the Theory of Planned Behaviour [13], Self Efficacy Theory [14], Intrinsic and Extrinsic Motivation and Self Determination Theory [15].

Application of expectancy-value theories has demonstrated that women's motivation to sustain breastfeeding behaviour at key points in the learning trajectory is challenged when they begin to experience negative breastfeeding attitudes (value) and believe that they cannot succeed (low expectancy for success). Conversely, women who sustain breastfeeding are found to be those who value breastfeeding and remained confident they can succeed. Early breastfeeding attrition researchers such as Janke [16] and Avery [17], although not referring directly in terms of value and expectancy for success, have provided breastfeeding researchers with key evidence that women who stopped breastfeeding early were also those whose initial intention was to breastfeed for much longer. One can therefore assume that although women's breastfeeding goal was at one point supported by an optimal level of both value for the behaviour and expectancy that they would be successful, some imbalance must have occurred that subsequently led to them to withdrawing from the behaviour.

As breastfeeding attrition is most commonly seen in first time mothers and is associated with a lack of support, a key psychological question that arises is: what is the motivational profile of first-time mothers who are breastfeeding? To answer this question we need to be able to define and measure maternal breastfeeding motivation using a valid and reliable instrument. We report on the sensitivity of a particular instrument, the Breastfeeding Motivational Measurement Scale (BMMS) [18] to detect the motivational constructs of first time mothers to sustain breastfeeding behaviour.

**Figure 1:**
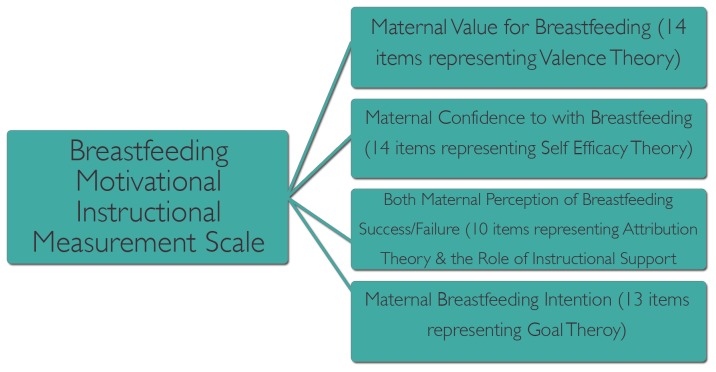
Theoretical Development of the BMM Scale. This figure provides a summary of the main theoretical components that were initially incorporated in the Breastfeeding Motivational Measurement Scale; including a measure of valence, self-efficacy, attributions that influence success or failure, and strength of intentions, or goal.

The BMMS was developed as a diagnostic tool, with the aim of measuring women's motivation for persisting to breastfeed while receiving routine instructional support by midwives. It contains 51 Likert-type items (7 point scale) that represented four theories, initially organized under five topics (Figure 1). A detailed description of the development process and completed instrument is available [18].

This paper investigates the construct validity of the BMMS tool by determining the motivational profile of first-time mothers after breastfeeding behaviour has been commenced.

## Methods

### Ethics Statement

No serious ethical concerns were identified and approval was obtained from the Office for Research Ethic Committees Northern Ireland. Governance approval was obtained from the South Eastern Health and Social Care Trust.

### Participants

Women were invited to participate whether they were first-time mothers or had a previous baby, whether they sustained breastfeeding or not. Included were women who initiated breastfeeding (giving at least one breastfeed) and were receiving instructional support from a midwife either in hospital or the community. Women who had breastfed within the previous four hours, but had since indicated their intention to stop breastfeeding were included if formula feeding had not yet commenced. We excluded non-English speaking women, incidences of infant and mother separation and infant abnormality known to complicate breastfeeding. The midwives acted as gatekeepers and they identified suitable participants to take part in the study and confirmed eligibility. Following confirmation by the community midwives of the appropriateness of contacting women identified within the community, the researcher made initial contact by telephone, providing verbal information about the study purpose and an information sheet detailing the structured interview process associated with completion of the questionnaire (BMMS). Permission for a visit (either at home or in hospital), was obtained and following written consent, an interview was conducted at a time that suited the mother. A pilot study (n = 20) was completed by a convenience sample of women in the postnatal environment, who had commenced breastfeeding.

### Sample

Sample size for the principle components analysis was determined by Gorsuch [19] rule of 200. All women identified gave permission to participate (n = 182). As no structural changes were made to the questionnaire following the pilot study (n = 20), the two samples were pooled, resulting in a final sample size of 202 women. Pooling samples to increase sample size in relation to factor analysis can be advantageous [20].

## Analysis and Results

Data analyses were completed in three stages: stage one comprised of data cleaning processes, stage two explored the higher level factor structure in relation to all mothers, stage three explored the factor structure related to first time mothers only.

### Stage one: data cleaning processes

Following data entry into SPSS (vs 11.5), Kaiser's Measure [21] of sampling adequacy (0.900), indicated that the items were appropriate for Principle Components Analysis (PCA) [20]. As the relationship between the theoretical measures of goals, self-efficacy, value and attributions had not previously been explored in relation to breastfeeding duration, exploratory factor analysis was used to explore the factor structure associated with early breastfeeding behaviours. Given that factor analysis is particularly sensitive to outlying cases [20], [24], outliers were detected using hierarchal clustering analysis. Initially two cases furthest from the cluster (cases 24 and 160) were identified and removed, and case 156 emerged as a further outlier. When these three outliers were de-selected and discriminate analysis repeated, no further outliers were evident in the sample. Complete data were available for 188 participants; an univariate and missing value analysis was completed that indicated that less than 5% of values per variable were missing. The results of the missing analysis were reviewed by the research team (including the senior statistician and theorist) and it was agreed that the findings did not demonstrate any missing values pattern of concern. In line with the statistical experts [20] (p 63), regression was used by the senior statistician to impute 11 missing values, giving a final sample size of 199 (99 FTM, 100 EM) for factor analysing.

### Stage 2: Higher level factor structure of all mothers

Cattell & Schuerger [22] acknowledged that use of eigenvalues alone can result in an overestimation of factors, hence the main criteria used to decide on the number of meaningful factors was based on (a) Kaiser's (1960) [21] eigenvalues >1 factor extraction rule, (b) scree plot analysis and (c) expert interpretability of the resulting factor structure [20]. This is the standard approach to exploratory factor analysis. PCA with Oblimin rotation was completed using all mothers in the first analysis and the scree plot was used to identify the main factors.

Two hundred and two women who were receiving routine postnatal breastfeeding instruction were approached and gave consent to complete the BMMS. Of this sample, 100 were first-time mothers. One hundred and sixty-six were interviewed while in hospital and the remaining 36 in the community. The mean age for the total sample was 30 years of age. Twenty per cent of women reported that they did not work, 26% reported having a professional vocation, 12% a managerial vocation, 18% clerical, 11% skilled and 13% non-skilled or other. Overall 28 women (14%) had already made the decision to discontinue breastfeeding and so intended to commence formula feeding within hours of completing the interview (of which 16 were first time mothers).

**Figure 2:**
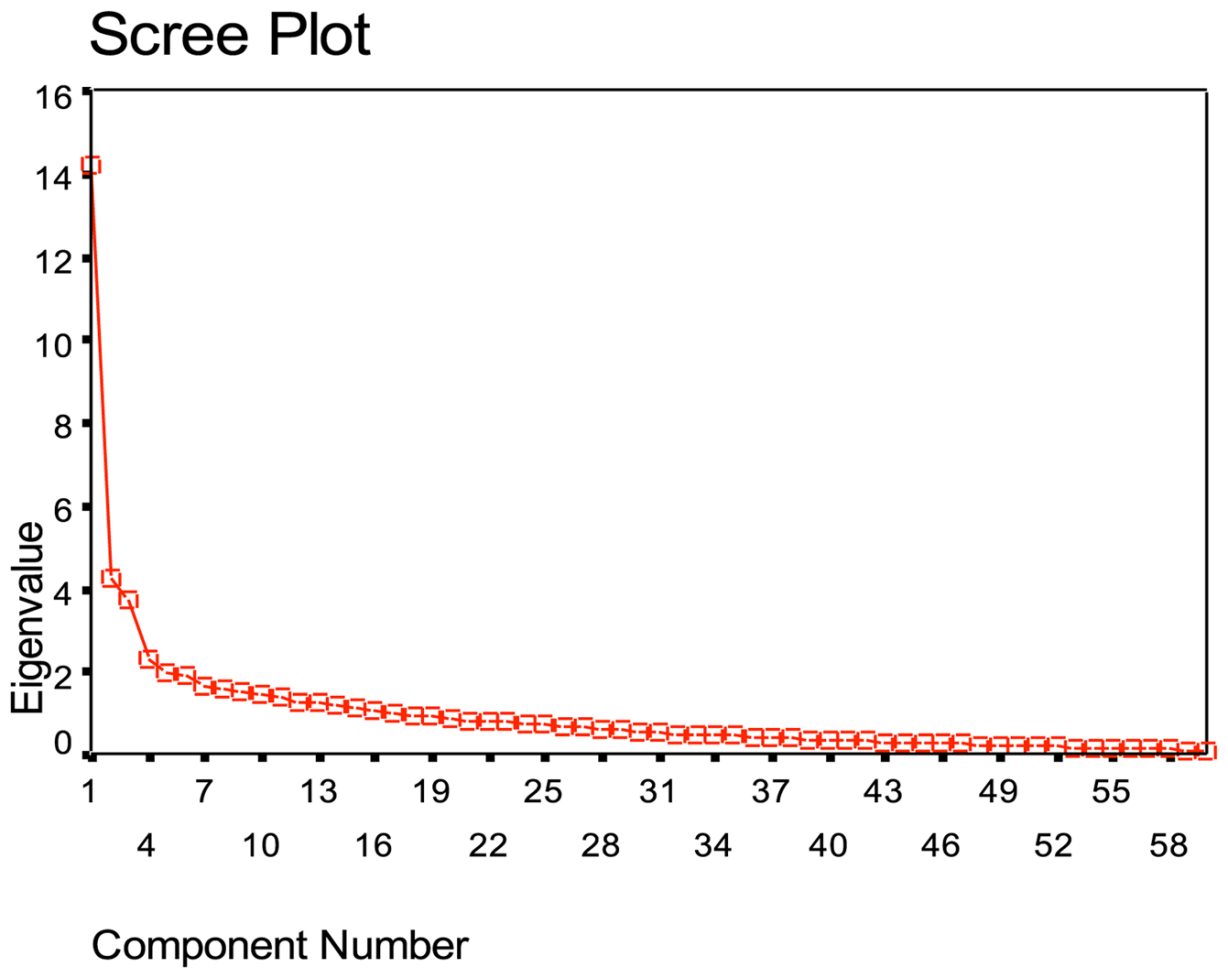
Scree Plot of the BMM Scale. This figure depicts the scree plot associated with stage two of the analysis: PCA (with Oblimin) using first time mothers and experienced mothers.

In total, 51 items loaded onto the eleven factors that explained 70% of the variance; however factors 5 to 11 had between 3 and 1 factor loadings and so were considered to be un-interpretable by the expert team. Considering that direct Oblimin rotation may produce higher eigenvalues, the team reviewed the eigenvalues for the remaining factors 1–5. Taking into consideration the scree plot (Figure 2) and considering the theoretical interpretability of the factors, the expert team agreed that the cut off point for factor rotation was three. A three factor solution was therefore accepted. This cut off was supported by the eigenvalues for factors 1 to 3, which ranged from 9.64 to 3.047. In relation to interpretability, although four expectancy-value theories had originally been incorporated into the scale, the initial analysis resulted in a 3-factor solution.

### Stage three: Exploring First Time Mothers as a Known Group

To assist with interpretation and in step with Goddard and Kirby [23] and Tabachnick and Fidell [20] factoring was attempted using an known-group approach. It was hypothesized that previous maternal experience would have an effect on women's expectancy for success and breastfeeding persistence. To avoid contamination of prior mothering experience, the known group analysis explored the factor structure associated with first time mothers. This again resulted in three factors and explained 49% of the variance, but the underlying structure remained hidden.

A component of several of the factors was identified as the self-efficacy scale; to avoid suboptimal factoring (bloated factors) and consequential masking of interpretable motivational constructs, the effects of the self-efficacy sub-scale were further explored. On agreement, PAF with Oblimin rotation, using three factors (cut off at .40) was used with the self-efficacy scale removed. The findings when the self-efficacy scale was removed explained 46% of the variance in first time mothers and reflected the overall value-expectancy structure in the pattern (Table 1) and factor score (Table 2) matrices. Supporting descriptive outputs for the Likert questions (File S1) and factor correlation outputs (File S2) are provided. Substantial internal consistency for these three factors was found; total value of breastfeeding (α = 0.96); perceived midwife support (α = 0.85) and expectancy to succeed (α = 0.84). An examination of these data from first-time mothers demonstrates a statistical three-factor model with theoretically meaningful constructs.

**Table 1:** Pattern Matrix of First Time Mothers using Principle Axis Factoring after Removing the Self Efficacy Scale.

BMMS Items	Factor 1 - Value	Factor 2 – MW Support	Factor 3 – Expect to Succeed
Breastfeeding is important to me	.621		
I would be upset if I did not manage to breastfeed	−.634		
The amount of time I spend breastfeeding keeps me from doing other things I would like to do			
The amount of effort I put into breastfeeding is worthwhile to me	.662		
Breastfeeding is not that important to me in the broad scheme of things	.639		
I like breastfeeding	.565		−.459
I don't like breastfeeding but I do it because it is the best way to feed my baby			−.421
Breastfeeding is very meaningful to me	.823		
I have considerable independence and freedom as to how I manage breastfeeding			−.577
I feel I cannot use my judgement when breastfeeding			−.580
Generally speaking I am very satisfied breastfeeding			−.709
I hate breastfeeding	.502		−.419
I feel a great sense of satisfaction when I breastfeed	.732		
I frequently think of quitting breastfeeding			−.735
My opinion of myself goes up when I breastfeed well	.552		
Overall I am no good at breastfeeding			−.676
I look forward to breastfeeding	.445		−.549
Overall I have a lot to be proud off	.402		
Breastfeeding requires me to learn skills through effort over time	.496		
I feel that I should personally take the credit or the blame for how breastfeeding goes			
My own feelings are generally not affected much one way or the other by how well I breastfeed			
Whether or not I breastfeed successfully is clearly my responsibility			
Most people who breastfeed feel a great sense of personal satisfaction			
I recieve lots of support and guidance from my midwives		.743	
The feedback I recieve from the midwives tells me what I want to know		.905	
There are things I would like to know about my breastfeeding experience that I am not being told		.698	
There are obvious challenges that I need to meet to breastfeed successfully			
The midwives let me know how well I am breastfeeding		.812	
I have a clear breastfeeding goal in mind	.542		
It is very important to me that I know how to work at reaching my breastfeeding goal	.637		
I can find out how good breastfeeding is going just by doing it			
As a result of feedback from my midwives I know I am breastfeeding well		.678	
Breastfeeding itself provides little information as to how well it is going			
The feedback I get from my midwives is not very useful		.673	
Breastfeeding is quite simple and repetitive			−.406
I have trouble figuring out whether breastfeeding is going well or not			−.638
I learn most things quickly			

Extraction Method: Principal Axis Factoring. Rotation Method: Oblimin with Kaiser Normalization.

a. Rotation converged in 20 iterations.

b. Only cases for which parity = prims are used in the analysis phase.

**Table 2:** Factor Score Coefficient Matrix of First Time Mothers using Principle Axis Factoring after Removing the Self Efficacy Scale.

BMMS Items	Factor 1 - Value	Factor 2 – MW Support	Factor 3 – Expect to Succeed
Breastfeeding is important to me	.134	.020	.069
I would be upset if I did not manage to breastfeed	−.059	.047	−.009
The amount of time I spend breastfeeding keeps me from doing other things I would like to do	.024	.000	.000
The amount of effort I put into breastfeeding is worthwhile to me	.100	.051	.003
Breastfeeding is not that important to me in the broad scheme of things	.038	.020	−.020
I like breastfeeding	.071	−.018	−.126
I don't like breastfeeding but I do it because it is the best way to feed my baby	.011	−.006	−.026
Breastfeeding is very meaningful to me	.304	−.071	.081
I have considerable independence and freedom as to how I manage breastfeeding	.041	−.028	−.042
I feel I cannot use my judgement when breastfeeding	−.060	.048	−.118
Generally speaking I am very satisfied breastfeeding	−.053	.113	−.228
I hate breastfeeding	.010	.044	−.108
I feel a great sense of satisfaction when I breastfeed	.170	−.008	.089
I frequently think of quitting breastfeeding	−.010	−.007	−.268
My opinion of myself goes up when I breastfeed well	.066	.030	.031
Overall I am no good at breastfeeding	.028	.011	−.104
I look forward to breastfeeding	.060	−.041	−.044
Overall I have a lot to be proud off	.049	.032	.001
Breastfeeding requires me to learn skills through effort over time	.108	.030	.149
I feel that I should personally take the credit or the blame for how breastfeeding goes	−.028	−.054	−.023
My own feelings are generally not affected much one way or the other by how well I breastfeed	−.013	−.024	.005
Whether or not I breastfeed successfully is clearly my responsibility	.013	.003	.062
Most people who breastfeed feel a great sense of personal satisfaction	.018	.038	−.026
I recieve lots of support and guidance from my midwives	.033	.111	−.022
The feedback I recieve from the midwives tells me what I want to know	−.131	.465	−.001
There are things I would like to know about my breastfeeding experience that I am not being told	.002	.114	−.001
There are obvious challenges that I need to meet to breastfeed successfully	−.005	.001	.037
The midwives let me know how well I am breastfeeding	.073	.245	.093
I have a clear breastfeeding goal in mind	.069	−.025	.028
It is very important to me that I know how to work at reaching my breastfeeding goal	.109	.069	.054
I can find out how good breastfeeding is going just by doing it	.018	.001	.060
As a result of feedback from my midwives I know I am breastfeeding well	.005	.086	−.002
Breastfeeding itself provides little information as to how well it is going	.015	.014	−.018
The feedback I get from my midwives is not very useful	.023	.043	.009
Breastfeeding is quite simple and repetitive	.013	−.019	.016
I have trouble figuring out whether breastfeeding is going well or not	−.053	−.025	−.189
I learn most things quickly	.052	.020	−.011

Extraction Method: Principal Axis Factoring. Rotation Method: Oblimin with Kaiser Normalization. Factor Scores Method: Regression.

a. Only cases for which parity = prims are used in the analysis phase.

## Discussion

First time mothers are more at risk of stopping breastfeeding in the early weeks, while previous maternal experience is known to have a positive effect on breastfeeding persistence and duration [25]–[26]. Likewise, instructional support is seen to positively influence breastfeeding outcomes [27]. Insight into the motivational profile of first time mothers during the early weeks of breastfeeding behaviour would enable a more targeted use of support-based resources, including insight into when expectancy-increasing strategies would be most effective. This known group analysis demonstrates the internal validity of the Breastfeeding Motivational Scale to identify the theoretical factors that represent first-time mothers' (FTMs) motivation to sustain the behaviour. However in order to further test the reliability and validity of the scale in replicate studies, this discussion focuses on the factoring process and theoretical importance of the findings as a framework for understanding first time mothers' motivation to sustain breastfeeding.

### Achieving a Balanced, Meaningful Solution

When conducting factor analysis, the main challenge that faces researchers is to extract the right number of factors that explains the maximum amount of variance, while at the same time providing a meaningful and interpretable solution. Recognising that exploratory factor analysis is a “complex procedure with few absolute guidelines and many options” [28] (p1), stage one of this analysis commenced with the application of PCA using Oblimin rotation; this resulted in an initial solution that although explained 70% of variance, produced 11 un-interpretable factors. Having identified the presence of three distinct factors in the initial scree plot (Figure 2), the systematic process for achieving the optimal solution as recommended in key texts [20], [28] was followed. This included using a factoring approach that would partition the unique variance from that shared, while maintaining the rotational approach recommended for use within the complexity of a social science investigation (Principle Axis Factoring PAF, using Oblimin rotation). However, in respect to finding a solution that would be also meaningful, Costello and Osborne (2005) [28] recommend that consideration should be given to what was already known about the population of interest. As first time mothers represent those most likely to stop breastfeeding, it was decided at this point in the analysis that first time mothers should be explored as a known group. The results of this next step in the factoring process saw the amount of variance drop considerably from 70% to 49%; however of more concern was the failure again to secure a meaningful solution. Keeping in mind that factor analysis experts advise that if a factor structure remains hidden after multiple factoring runs, then the researchers may have to recognise that the problem lies with the scale construction and design and that the data itself may most probably be unusable [28]. With this in mind, it was decided that PAF with Oblimin should be repeated again, however this time the self-efficacy items should be removed on the basis that these items could cause sub-optimal factoring. Removing the self-efficacy items could be defended theoretically in that the first time mothers may not yet have had sufficient breastfeeding experience to satisfy this main source of self-efficacy. On completion of this final analysis, the results maintained a three factors solution, explained 46% of variance and was both interpretable and meaningful. Four expectancy-value theories had originally been incorporated into the scale, however the three factors identified, directly reflected an expectancy-value structure that was influenced by midwifery support; first time mothers' value for breastfeeding, their perceived support from the midwives and their expectancy to succeed. On completion of the factoring process it was concluded that the best factoring solutions are achieved when researchers are guided by best practice and expertise in relation to research, statistics and theory that are brought to bear on the analytic process.

### The Theoretical Importance of the Resulting Three Factor Solution

Interpretation of the three factors presented in the pattern matrix for first time mothers (following removal of the self-efficacy items) demonstrated that this group of women valued breastfeeding and placed importance on midwife support, but experienced lower expectancy for success. Theorists have long recognised that there is an interrelatedness that exists between value for a behaviour and expectancy that you can succeed. According to Worrell [29], expectancies are known to remain significantly lower in a highly-valued situation; that is, when a behaviour is considered highly valuable and yet unattainable, this motivational imbalance is known to have a negative effect on the person's perceived experience and willingness to persist. It therefore follows that if first time mothers demonstrate a high value for breastfeeding coupled with a low expectancy for success, this motivational profile would represent an imbalance that is more likely to result in maternal feelings of anxiety, stress, breastfeeding dissatisfaction and cessation. This relationship between experience and willingness to persist is supported by Bandura's Theory of Self-Efficacy [14] wherein, experience that is perceived as negative is likely to create emotions such as anxiety or stress. These feelings are known to have a deleterious effect on maternal confidence (expectancy for success), and thus to negatively impact the person's willingness to persist and engage in the behaviour. Many breastfeeding researchers have reported on the maternal stress and anxiety experienced by first-time mothers in the lead up to breastfeeding cessation [30]–[33]. According to psychologists Martin and Tesser [34] (p45) the source of stress is the result of an unexpected aspect of an experience; it therefore follows that unexpected experiences can challenge a person's self-efficacious beliefs that they can succeed. However, an important point in this factoring process, was that the most interpretable solution emerged only after the self-efficacy scale was removed. Further research is required to explore this phenomenon, however the findings suggest that there may be an unknown trajectory associated with how first time mothers' expectancy for success beliefs develop throughout their breastfeeding experience. Discovering how first time mothers' motivation to breastfeed develops will enable the provision of more women-centred instructional support that can empower women to deal with the emotional complexity of learning to breastfeed [35]–[38]. From a theoretical perspective, psychologists such as Jacobs and Eccles [39] and Deci and Ryan [40] have provided compelling evidence that self-competency and autonomy are important achievement motives that enable individuals to make meaningful choices and when, individuals perceive themselves to be self-determined, they are more likely to freely process their personal learning needs in a given context and so spontaneously generate their actions in response.

## Conclusion

Theorists propose that optimal motivation consisting of high value and positive expectancies for success are required for behavioural persistence and achievement to occur when faced with challenging tasks [41]. In contrast, high value coupled with low expectancies for success is associated with low persistence and achievement in learners [42]. The results of the known group analysis demonstrate that first time mothers' motivation to sustain breastfeeding is related to the value they place on the behaviour and their expectancy to succeed, in relation to midwife support. Although the results demonstrate the theoretical validity of the BMMS, it also highlights the importance of applying theory as a means of monitoring the psychology that underpins breastfeeding behaviour. Findings from this study and future applications of this scale will inform behavioural psychologists, health educators and policy makers of the importance of theory-based and women-centred instructional design applied to antenatal and postnatal education. This questionnaire may be reproduced without permission however the authors must be cited in relation to any application of the tool or application of any transcribed version.

## Supporting Information

File S1 Descriptive Output for Likert Items in Stage 3 Analysis.
(DOCX)

File S2 The KMO, Factor Correlation and Covariance Matrices Related to Stage 3 of the Analysis.
(DOCX)
