# Correction: Sensitivity of the Breastfeeding Motivational Measurement Scale: A Known Group Analysis of First Time Mothers

**DOI:** 10.1371/journal.pone.0091382

**Published:** 2014-02-27

**Authors:** The PLOS ONE Staff

There are errors in the affiliations. The correct affiliations are listed below.

Janine Stockdale^1^*, Marlene Sinclair^1^, George Kernohan^1^, Evie McCrum-Gardner^2^, John Keller^3^

1 Institute of Nursing and Health Research, University of Ulster, Belfast, Northern Ireland, 2 Clinical Research Support Centre, Belfast, Northern Ireland, 3 Instructional Systems Faculty, Florida State University, Tallahassee, Florida, United States of America

* E-mail: dj.stockdale@ulster.ac.uk
