# Supplementary material for: Sensitivity of the Breastfeeding Motivational Measurement Scale: A Known Group Analysis of First Time Mothers
Source: PLoS One. 2013 Dec 31;8(12):e82976. doi: 10.1371/journal.pone.0082976 (PMC3876990; doi:10.1371/journal.pone.0082976)
Supplement: File S1 — Descriptive Output for Likert Items in Stage 3 Analysis. (DOCX) [file pone.0082976.s001.docx]

Descriptive Output for Likert Items in Stage 3 Analysis S1

| **Descriptive Statistics** | | | | | | |
| --- | --- | --- | --- | --- | --- | --- |
|  | N | Range | Minimum | Maximum | Mean | Std. Deviation |
| Breastfeeding is important to me | 199 | 5.00 | 2.00 | 7.00 | 6.3869 | .70784 |
| I would be upset if I did not manage to breastfeed | 199 | 6.00 | 1.00 | 7.00 | 2.2814 | 1.44991 |
| The amount of time I spend breastfeeding keeps me from doing other things I would like to do | 199 | 6.00 | 1.00 | 7.00 | 4.7337 | 1.80191 |
| The amount of effort I put into breastfeeding is worthwhile to me | 198 | 6.00 | 1.00 | 7.00 | 6.2323 | .89361 |
| Breastfeeding is not that important to me in the broad scheme of things | 199 | 6.00 | 1.00 | 7.00 | 5.5678 | 1.44745 |
| I like breastfeeding | 198 | 6.00 | 1.00 | 7.00 | 5.6717 | 1.41719 |
| I don't like breastfeeding but I do it because it is the best way to feed my baby | 198 | 6.00 | 1.00 | 7.00 | 5.3586 | 1.67019 |
| Breastfeeding is very meaningful to me | 199 | 6.00 | 1.00 | 7.00 | 5.7286 | 1.24994 |
| I have considerable independence and freedom as to how I manage breastfeeding | 198 | 6.00 | 1.00 | 7.00 | 4.7071 | 1.85928 |
| I feel I cannot use my judgement when breastfeeding | 198 | 6.00 | 1.00 | 7.00 | 5.1919 | 1.71690 |
| Generally speaking I am very satisfied breastfeeding | 199 | 6.00 | 1.00 | 7.00 | 5.0854 | 1.82235 |
| I hate breastfeeding | 199 | 6.00 | 1.00 | 7.00 | 6.2211 | 1.19821 |
| I feel a great sense of satisfaction when I breastfeed | 199 | 6.00 | 1.00 | 7.00 | 6.2613 | 1.12009 |
| I frequently think of quitting breastfeeding | 198 | 6.00 | 1.00 | 7.00 | 4.6566 | 2.03104 |
| My opinion of myself goes up when I breastfeed well | 199 | 6.00 | 1.00 | 7.00 | 5.4824 | 1.55324 |
| Overall I am no good at breastfeeding | 199 | 6.00 | 1.00 | 7.00 | 5.2714 | 1.87399 |
| I look forward to breastfeeding | 199 | 6.00 | 1.00 | 7.00 | 4.9698 | 1.74051 |
| I learn most things quickly | 199 | 6.00 | 1.00 | 7.00 | 5.9397 | .99817 |
| Overall I have a lot to be proud off | 199 | 6.00 | 1.00 | 7.00 | 6.1709 | 1.05455 |
| Breastfeeding requires me to learn skills through effort over time | 199 | 6.00 | 1.00 | 7.00 | 5.8643 | 1.51647 |
| I feel that I should personally take the credit or the blame for how breastfeeding goes | 199 | 6.00 | 1.00 | 7.00 | 3.7035 | 1.86340 |
| My own feelings are generally not affected much one way or the other by how well I breastfeed | 198 | 6.00 | 1.00 | 7.00 | 2.7121 | 1.81728 |
| Whether or not I breastfeed successfully is clearly my responsibility | 199 | 6.00 | 1.00 | 7.00 | 4.4623 | 1.87685 |
| Most people who breastfeed feel a great sense of personal satisfaction | 199 | 6.00 | 1.00 | 7.00 | 6.1608 | 1.08445 |
| I recieve lots of support and guidance from my midwives | 199 | 6.00 | 1.00 | 7.00 | 5.6683 | 1.44269 |
| The feedback I recieve from the midwives tells me what I want to know | 199 | 6.00 | 1.00 | 7.00 | 5.2010 | 1.80062 |
| There are things I would like to know about my breastfeeding experience that I am not being told | 199 | 6.00 | 1.00 | 7.00 | 4.6985 | 1.93050 |
| There are obvious challenges that I need to meet to breastfeed successfully | 197 | 6.00 | 1.00 | 7.00 | 5.3553 | 1.48311 |
| The midwives let me know how well I am breastfeeding | 199 | 6.00 | 1.00 | 7.00 | 4.8593 | 1.74376 |
| I have a clear breastfeeding goal in mind | 199 | 6.00 | 1.00 | 7.00 | 5.4623 | 1.53982 |
| It is very important to me that I know how to work at reaching my breastfeeding goal | 198 | 6.00 | 1.00 | 7.00 | 5.4394 | 1.37928 |
| I can find out how good breastfeeding is going just by doing it | 197 | 6.00 | 1.00 | 7.00 | 2.7766 | 1.55867 |
| As a result of feedback from my midwives I know I am breastfeeding well | 199 | 6.00 | 1.00 | 7.00 | 4.5226 | 1.90646 |
| Breastfeeding itself provides little information as to how well it is going | 198 | 6.00 | 1.00 | 7.00 | 4.4141 | 1.85522 |
| The feedback I get from my midwives is not very useful | 199 | 6.00 | 1.00 | 7.00 | 4.9497 | 1.92209 |
| Breastfeeding is quite simple and repetitive | 199 | 6.00 | 1.00 | 7.00 | 3.3216 | 1.76872 |
| I have trouble figuring out whether breastfeeding is going well or not | 198 | 6.00 | 1.00 | 7.00 | 4.1010 | 1.98213 |
| Valid N (listwise) | 194 |  |  |  |  |  |
