# Supplementary material for: Sensitivity of the Breastfeeding Motivational Measurement Scale: A Known Group Analysis of First Time Mothers
Source: PLoS One. 2013 Dec 31;8(12):e82976. doi: 10.1371/journal.pone.0082976 (PMC3876990; doi:10.1371/journal.pone.0082976)
Supplement: File S2 — The KMO, Factor Correlation and Covariance Matrices Related to Stage 3 of the Analysis. (DOCX) [file pone.0082976.s002.docx]

The KMO, Factor Correlation and Covariance Matrices Related to Stage 3 of the Analysis S2

| **KMO and Bartlett's Test^a^** | | |
| --- | --- | --- |
| Kaiser-Meyer-Olkin Measure of Sampling Adequacy. | | .783 |
| Bartlett's Test of Sphericity | Approx. Chi-Square | 1997.366 |
|  | df | 630 |
|  | Sig. | .000 |
| a. Only cases for which parity = prims are used in the analysis phase. | | |

| **Factor Score Covariance Matrix^a^** | | | |
| --- | --- | --- | --- |
| Factor | 1 | 2 | 3 |
| 1 | .935 | .456 | 1.651 |
| 2 | .456 | 1.076 | .065 |
| 3 | 1.651 | .065 | 2.714 |
| Extraction Method: Principal Axis Factoring.  Rotation Method: Oblimin with Kaiser Normalization.  Factor Scores Method: Regression.^a^ | | | |
| a. Only cases for which parity = prims are used in the analysis phase. | | | |

| **Factor Correlation Matrix^a^** | | | |
| --- | --- | --- | --- |
| Factor | 1 | 2 | 3 |
| 1 | 1.000 | .192 | -.162 |
| 2 | .192 | 1.000 | -.157 |
| 3 | -.162 | -.157 | 1.000 |
| Extraction Method: Principal Axis Factoring.  Rotation Method: Oblimin with Kaiser Normalization.^a^ | | | |
| a. Only cases for which parity = prims are used in the analysis phase. | | | |
